# Training, Practice and Service Provision for Selective Mutism: Findings From a UK‐Wide Survey of Therapists

**DOI:** 10.1111/1460-6984.70297

**Published:** 2026-08-10

**Authors:** Gino Hipolito, Rachel Batchelor, Victoria Joffe, Kate Nation, Cathy Creswell

**Affiliations:** ^1^ Paediatric Speech and Language Therapy Department St George's University Hospitals NHS Foundation Trust London UK; ^2^ Department of Experimental Psychology University of Oxford Oxford UK; ^3^ School of Health and Social Care University of Essex Colchester UK; ^4^ Department of Psychiatry University of Oxford Oxford UK

**Keywords:** intervention, selective mutism, survey, therapists, training

## Abstract

**Background:**

Selective mutism (SM), an anxiety disorder often beginning in early childhood, can impair communication, social interaction, and educational progress. When left untreated, problems may persist into adulthood and impact longer term mental health. Although effective interventions exist, many children with SM are unable to access support services, in part due to the lack of suitable training for professionals in SM, the absence of national guidelines, and unclear professional responsibilities which together create gaps in provision.

**Aim:**

To survey UK therapists working in the National Health Service (NHS), Local Authorities or Health and Social Care (HSC) services who support preschool and primary school‐aged children (3–12 years) with SM to identify the training therapists have received, their perceived training needs, the professions providing interventions, and the nature of the interventions delivered.

**Methods:**

A 32‐item online survey was distributed via professional organisations and specialist networks, charity forums, and social media using snowball sampling. Results were analysed using descriptive statistics and free text quotes depicting therapists’ opinions or experiences were used to triangulate the quantitative data.

**Results:**

Of 244 responses, 201 met eligibility criteria. Most respondents were speech and language therapists (81%), followed by clinical psychologists (11%), educational psychologists (5%) and other professions (3%). Just over half (59%) had received some form of SM training, but only 15% received training during their professional qualification. The majority (85%) expressed the need for additional training in SM to do their jobs particularly in the areas of delivering intervention, addressing co‐occurring conditions and working within multidisciplinary teams. Three quarters of the sample (75%) had provided intervention for children with SM, predominantly within schools, involving parents/carers (95%) and teaching staff (96%). Intervention complexity and number of components increased with child age; the most common components across ages were exposure, rapport building, transfer of control and psychoeducation. However, there was extensive variability in the dosage of the interventions provided.

**Conclusions and Implications:**

This exploratory survey with a non‐representative sample suggested that the intervention components used, the people involved, and where the intervention occurred generally reflected the available evidence base. However, even in this sample who are more likely to have an interest in SM, there are still gaps in professional training and variability in service provision for children with SM. To promote consistent, evidence‐based care, we recommend development of national cross‐profession guidelines and quality standards for professional training and clinical management of children with SM.

**WHAT THIS PAPER ADDS:**

*What is already known on the subject*

- Effective interventions for selective mutism (SM) exist, but many children cannot access appropriate support. Inadequate professional training, absence of national guidelines, and unclear responsibilities across professions have been identified as key barriers to provision.

*What this paper adds to the existing knowledge*

- This first UK‐wide exploratory survey of therapists highlights that SM training is rarely included in professional qualification courses, leaving most practitioners seeking additional training post‐qualification. Intervention components used by the sample of therapists reflected the current evidence base and increased in complexity with age; there was wide variation in dosage and delivery. The findings provide new evidence on which professions deliver SM interventions in a sample of UK therapists and the training gaps they experience.

*What are the potential implications of this study?*

- There is a pressing need to embed SM training into professional qualification programmes and to provide accessible cross‐professional specialist training post‐qualification. Developing cross‐profession guidelines and quality standards will help ensure consistency, accountability and equitable delivery of evidence‐based interventions for children with SM across the UK.

## 1 Background

Selective mutism (SM) is an anxiety disorder in which an individual is unable to speak in specific situations where speech is expected (e.g. to teachers in class) despite being able to speak in other situations (e.g. to parents at home) (American Psychiatric Association 2013, World Health Organization 2022). To meet diagnostic criteria this pattern of mutism should last for over a month and not be due to a lack of knowledge or comfort with the language or be better explained by another communication disorder or condition like autism, schizophrenia or another psychotic disorder (American Psychiatric Association 2013, World Health Organization 2022). The condition often first manifests between the ages of 2 and 5 years with prevalence figures ranging between 0.03% and 2% in the paediatric population (Bergman et al. 2002, Elizur and Perednik 2003, Karakaya et al. 2008, Kopp and Gillberg 1997, Kumpulainen et al. 1998, Sharkey and McNicholas 2012, Szczerbinski et al. 2019). Variation in figures reflects the type of the study, the age of children, and the different criteria used to identify SM (Hipolito and Johnson 2021). SM has a negative impact on children's education and interactions with others (Bergman et al. 2002, Kumpulainen et al. 1998) and is associated with high rates of emotional and behavioural problems during childhood (Kristensen 2001, Steinhausen and Juzi 1996). These mental health problems and difficulties with communication during education, leisure activities, and work often continue into adulthood (Remschmidt et al. 2001, Steinhausen et al. 2006).

SM is treatable (Hipolito et al. 2023), particularly when identified early (Stone et al. 2002). A recent systematic review and meta‐analysis found that combined systems interventions (targeting the knowledge, skills, and interactions of significant people around the child with SM; Zakszeski and DuPaul 2017) and behavioural interventions (targeting behavioural factors thought to maintain the mutism, e.g. avoidance) were promising for improving both speaking behaviour and SM remission in children aged 3–9 years (Hipolito et al. 2023). The review analysed 25 nonpharmacological intervention studies targeting SM. The most common components used in interventions for SM were exposure activities (through prompting, graded exposure shaping, video modelling and/or stimulus fading), positive reinforcement/reward systems (e.g. quiet praise, tangible reinforcers such as reward charts or activity reinforcers like screen time), psychoeducation about SM, and rapport building strategies (e.g. defocused communication, triangle tactic; Oerbeck et al. 2012, Johnson and Wintgens 2016). Six other components that were also identified were used in less than half of the studies (coping strategies, transfer of control, social skills, cognitive strategies, problem solving and play therapy).

Although effective interventions exist, many parents of children with SM are not able to access support services (Hipolito and Creswell 2024). During a Patient Public Involvement activity that the authors conducted in the UK in 2021, 64% (*n* = 61/95 responders) of parents and carers of children with SM surveyed via the Selective Mutism Information and Research Association (SMIRA) Parent Facebook page indicated that they were not able to receive intervention for their child's SM from their Health Service or Local Authority.

Lack of clear guidance for treatment of SM to ensure consistency and accountability hinders access to effective treatments. The American Speech‐Language‐Hearing Association's (ASHA) practice portal and the Royal College of Speech and Language Therapists’ (RCSLT) website have clinical guidelines for SM (American Speech‐Language‐Hearing Association n.d., Royal College of Speech and Language Therapists 2018). However, other international professional associations in speech and language therapy (Irish Association of Speech and Language Therapists, Speech Pathology Australia) or educational psychology (National Association of School Psychologists, NASP, USA) have information, handouts or resources about SM but no specific clinical guidelines for SM. Other psychology (Society of Clinical Child & Adolescent Psychology, USA) and psychiatry (American Academy of Child and Adolescent Psychiatry) professional associations subsume SM under the broader anxiety disorders guidelines (Society of Clinical Child and Adolescent Psychology 2017, Connolly and Bernstein 2007, Walter et al. 2020). Some years ago, Keen et al. (2008) used a consensus of international and interprofessional expert opinions to develop a care pathway of good practice for children with SM to compensate for the lack of evidence base at the time. The researchers recommended the development of UK guidelines for clinical management of children with SM. However, to this day there are no National Institute for Health and Care Excellence [NICE] guidelines for SM. Although the RCSLT has SM clinical guidelines for speech and language therapists to follow, to the authors’ knowledge, equivalent guidelines do not exist for other key professional groups in the UK.

Lack of training or inadequate training may be a barrier to applying the evidence base into clinical practice (Zipoli and Kennedy 2005, Greenwell and Walsh 2021) and thereby hinder access to effective treatments. Keen et al. (2008) raised concerns that existing training in key professional groups (speech and language therapists, educational, and clinical psychologists) were inadequate in Europe (including the UK), North America, and Australia. It is important to investigate the training key professional groups are receiving to support children with SM.

There are very few surveys exploring professionals’ training and experience with SM. Ellis (2015) surveyed 166 school psychologists (SP) (educational psychologists) in California, USA, and found 71.5% (*n* = 118) did not receive any training in SM in their pre‐registration programme and 90.3% (*n* = 149) felt inadequately prepared to help SM students in their schools after graduating. Williams (2019) surveyed 102 school‐based speech and language pathologists (SLP) and 79 SP in the USA about their knowledge and role in working with children with SM. Despite the respective national professional bodies (ASHA and NASP) advocating for their professions’ responsibility to assess and treat children with SM, 21% of SLPs (*n* = 21) and 13% of SPs (*n* = 10) did not see treatment for SM as being within their scope of practice. Williams (2019) inferred that this incongruence indicated the need for proper training and continued education in SM for SLPs and SPs. The SM training received and training needs of therapists in the UK have not been investigated.

In the UK, like many other countries, providing interventions for children with SM is not the responsibility of one single profession. In Keen et al. (2008) Delphi study involving 13 recognised clinical and academic experts in the field of SM from Europe (including the UK), North America, and Australia, there was no consensus on which profession should take the lead in supporting a child with SM. This is in part due to the different roles speech and language therapists and educational and clinical psychologists play within and between countries represented in the Delphi consultation. They did, however, agree that each local service should identify which professionals take the lead in providing an initial intervention to prevent treatment delay. While this is a pragmatic resources‐ and needs‐led approach, it may still create gaps in provision if local services have not identified a lead profession. And as such, it is not clear which professions are currently offering interventions and what is usual practice. Therefore, it is important to know who is providing the intervention for children with SM, what they are providing, and whether it is in line with the current evidence base.

The aim of this study was to survey UK therapists who provided psychological therapy or speech and language therapy and worked in the National Health Service (NHS), Local Authorities, or Health and Social Care (HSC) (Northern Ireland) and who are most likely to see preschool and primary school aged children with SM to understand:

1. What SM‐related training are therapists receiving?
2. What are their training needs?
3. Who are the therapists providing interventions for SM?
4. What interventions are these therapists providing?

## 2 Methods

### 2.1 Ethics

The Joint Research Office study classification group at the University of Oxford reviewed the study and determined it to not be subject to the Department of Health's UK Policy Framework for Health and Social Care Research (2017). Therefore, the study did not require research ethics review. Prior to accessing the survey questions, therapists were asked to read the participant information sheet, give consent, and then answer the inclusion criteria questions.

### 2.2 Participants

The study used a non‐probability purposive sample of therapists who fulfilled the following criteria:

- Provided some form of psychological therapy or speech and language therapy,
- Worked with children 3 to 12 years old,
- Worked for or provided a service for the NHS, Local Authority or HSC services in the United Kingdom.

This population may have included but was not limited to speech and language therapists, clinical psychologists, educational psychologists, CAMHS workers, psychotherapists, play therapists, art therapists, music therapists, mental health nurse or health and well‐being practitioners.

### 2.3 Survey Design

The survey development was based on an adapted version of Wilson and Dickinson's Agile approach to survey development (Wilson and Dickinson 2022). The final protocol followed the CHERRIES statement (Checklist for Reporting Results of Internet E‐Surveys)(Eysenbach 2004) and was uploaded and registered on the Open Science Framework in February 2024 (https://doi.org/10.17605/OSF.IO/CTWFJ) The online survey was programmed using Qualtrics XM (2023) and consisted of a 32‐item questionnaire (see Supplementary Materials 1) covering the following areas to answer the study questions:

- Demographic information: profession, length of time in the profession, UK country of work (i.e. England, Northern Ireland, Scotland or Wales), age and highest level of education (5 questions).
- Therapists’ awareness of SM consisting of one self‐rating Likert scale (1–7) question and six questions about the nature of the condition (7 questions),
- Therapists’ training on SM: when they received it (i.e. during professional qualification, post‐qualification university courses, post‐qualification additional specialist training, inset/in‐house training), the name of the course, length of the course and perceived training needs (6 questions),
- Therapists’ approximate SM caseload in the last 5 years for assessment and intervention (2 questions),
- The SM interventions provided by therapists covering who was involved, where it occurred, the mode and format of the intervention, dosage and which components were used across the age ranges 3–5, 6–9 and 10–12 years (12 questions). Therapists chose from a list of ten components with access to definitions and an ‘other’ option with an open textbox. The ten components and definitions were derived from a recent systematic review on SM interventions (Hipolito et al. 2023) (see Supplementary Materials 2 for definitions used in the survey).

### 2.4 Recruitment

A recruitment flyer was shared with relevant professional organisations (Royal College of Speech and Language Therapists, British Psychological Society, Association of Educational Psychologists, British Association for Music Therapy, British Association of Art Therapists, British Association of Play Therapists), networks (Selective Mutism Clinical Excellence Network, NHS Therapist SM Support Group), charity online forums (Selective Mutism Information and Research Association, SMIRA), and through their communication channels (e.g. email). The flyer was also posted on social media (X). The organisations and participants were encouraged to share the survey with others in their networks who fulfilled the study criteria. Although this snowball sampling method increases the risk of bias, it can be effective in reaching hidden or rare populations (Faugier and Sargeant 1997). The survey was open for 16 weeks (November 2023 to February 2024).

### 2.5 Data Analysis

Prior to data analysis, a Data Integrity Protocol v4Feb24 (see Supplementary Materials 3) was applied by two coders (GH and RB). This removed entries that were ineligible, incomplete, or were at risk of being multiple entries, fraudulent responses, or online bots. As set‐out in the study protocol, completion rates were based on the ratio of participants who completed the survey (those who have completed 60% or more) divided by the number of people who gave consent to participate.

Due to the non‐probabilistic nature of snowball sampling, the distribution within occupation groups in the final sample will likely be non‐representative and therefore, inferential statistics were not used to compare groups. Instead, descriptive statistics were used for responses to closed‐ended questions. We originally planned to analyse free‐text responses of therapists’ opinions or experiences using inductive content analysis as described by Elo and Kyngäs (2008). However, the data did not allow for a full content analysis due to the limited statements recorded. Instead, we triangulated the qualitative responses with the quantitative data to illustrate the answers to the research questions (Kawar et al. 2024).

## 3 Results

Responses from 244 individuals were recorded in Qualtrics at the end of the survey period. The completion rate of the survey was 89% (217/244). The 27 incomplete responses consisted of 16 who did not fulfil the inclusion criteria and 11 who completed less than 60% of the survey. The Interrater Reliability of the two coders applying the Data Integrity Protocol v4Feb24 was ‘almost perfect’ (Cohen's Kappa = 0.94, 95% CI: 0.89–1.00). A total of 43 survey entries were removed (incomplete *n* = 27, at risk *n* = 16) resulting in the final sample of 201 participants for the data analysis.

From the sample, there were thirteen free text responses regarding a therapist's opinion or experience during the survey.

### 3.1 Demographics

The participant demographic details are presented in Table 1. Most of the participants were speech and language therapists (80.60%, *n* = 162), a tenth of the sample were clinical psychologists (10.95%, *n* = 22), followed by educational psychologists (4.98%, *n* = 10) (see Table 1). Several other professions (play therapists, music therapists, counselling psychologists, and community health nurses) were grouped into an ‘Other’ category to preserve anonymity (3.48%, *n* = 7).

**TABLE 1:** Participants demographic data.

Demographic category	Number	Percentage of sample
*Professional group*		
Speech and language therapists	162	80.60%
Clinical psychologists	22	10.95%
Educational psychologists	10	4.98%
Other (play therapists, music therapists, counselling psychologist, community mental health nurse)	7	3.48%

*UK country*		
England	114	56.72%
Scotland	58	28.86%
Wales	14	6.97%
Northern Ireland	15	7.46%

*Highest level of education*		
Pre‐registration undergraduate	85	42.29%
Pre‐registration postgraduate Diploma/masters	34	16.92%
Post‐registration Postgraduate Certificate/diploma	22	10.95%
Post‐registration masters	26	12.94%
Doctorate	34	16.92%

*Participant age*	*Mean (SD) years*	*Range*
*Whole sample*	*38.65 (9.40)*	*21*–*67*
Speech and language therapists	38.01 (9.76)	21–67
Clinical psychologists	40.68 (6.70)	30–57
Educational psychologists	40.90 (8.31)	31–56
Other	43.86 (8.09)	34–55

*Average years in profession*	*Mean (SD)*	*Range*
*Whole sample*	*11.34 (8.70)*	*0.16*–*37*
Speech and language therapists	11.33 (8.97)	0.16–37
Clinical psychologists	11.32 (6.28)	2–24
Educational psychologists	12.40 (9.28)	3–30
Other	10.14 (9.65)	1–30

*Note*: Whole sample is 201 participants.

Over half of the participants worked in England (56.72%, *n* = 114), just under a third in Scotland (28.86%, *n* = 58) and a smaller portion in Northern Ireland (7.46%, *n* = 15) and Wales (6.97%, *n* = 14).

Most respondents reported their highest level of education as ‘pre‐registration undergraduate’ (42.29%, *n* = 85), followed by ‘post‐registration postgraduate’ (23.88%, *n* = 48); ‘pre‐registration postgraduate’ and ‘doctorate’ level each comprised a sixth of the sample (16.92%, *n* = 34).

The mean age of the respondents was 38 years (SD = 9.40, range 21–67). The speech and language therapists mirrored the sample mean of 38 years (range 21–67 years), while the mean age of clinical psychologists (range 30–57 years) and educational psychologists (range 31–56 years) were 40 years. The ‘Other’ therapists were slightly older at 43 years (range 34–55 years).

Average time in their respective professions was 11 years (mode = 4) ranging from newly qualified up to 37 years of experience. Average clinical experience was similar for each of the professional groups, ranging between 10 and 12 years.

### 3.2 Research Question 1: What SM Training Are Therapists Receiving?

Fifty‐nine percent of the full sample (118/201) had received some form of training in SM. When divided into professional groups, only a few clinical psychologists had received training in SM (9%, *n* = 2). However, 60% (*n* = 6) of educational psychologists and 66% (*n* = 107) of speech and language therapists had received some form of training in SM, while 43% (*n* = 3) of the ‘Other’ professional group had received some training in SM.

**FIGURE 1:**
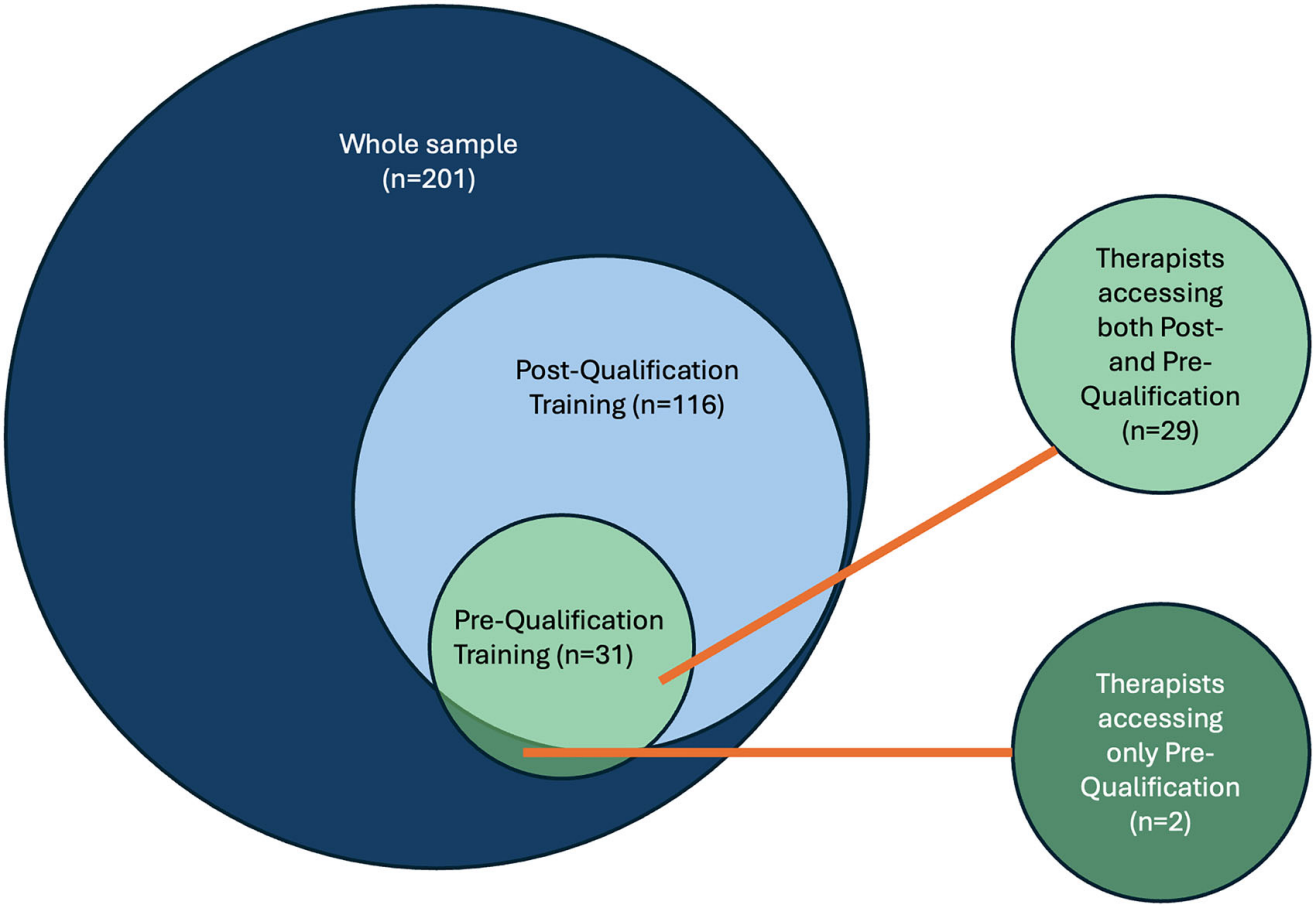
Participating therapists’ access to selective mutism training.

Figure 1 shows when the respondents accessed SM training; 57.71% (*n* = 116) accessed training post‐qualification while only 15.42% (*n* = 31) received training during their professional qualification. One respondent expressed feeling a sense of shock and pressure when faced with seeing a child with SM without training:

*We did not cover SM during my speech and language therapy training as far as I recall which was a shock when I took on my first SM child for therapy and had to try and read a large section of the Maggie Johnson book prior to seeing them* (Respondent 11).

The quotes in the results are attributed to the participant and a qualifying number that describes the order they completed the survey in relation to the sample (i.e. Respondent 1 refers to the first participant who completed the survey).

### 3.3 Research Question 2: What Are the Therapists’ Training Needs?

Most respondents (84.58%, *n* = 170) reported needing extra training in SM to do their job (see Table 2). This was consistent across the professional groups. The most commonly reported training need was ‘SM intervention’ (74.63%, *n* = 150), ‘working in multi‐disciplinary teams’ (55.72%, *n* = 112) and ‘intervention for SM with comorbid conditions’ (62.69%, *n* = 126) (see Table 3), in particular autism (41.27%, *n* = 52), language disorders (12.70%, *n* = 16), and speech sound disorders (7.94%, *n* = 10). In the ‘Other need’ category, three respondents wanted training in working with teenagers with SM, five made comments related to the earlier categories, and one wanted to learn time efficient ways for educational psychologists to work with children with SM. One respondent shared in the ‘Other need’ category that they would like to learn more from people with lived experiences to shape their practice:

*More awareness generally from people who are/have themselves been situationally mute and so can inform compassionate and accurate practice going forwards* (Respondent 171).

**TABLE 2:** Therapists providing assessment and intervention for children with selective mutism and their self‐reported need for extra training to do their jobs.

	Self‐reported need for extra selective mutism training				Providing assessment and/or intervention for children with selective mutism	Providing intervention for children with selective mutism
Participants	Degree of need	Number	Percentage	Total ‘yes’ Percentage (n)	Percentage (n)	Percentage (n)
Whole sample	Definitely yes	79	39.30%	84.58% (170)	93.03% (187)	75.12% (151)
	Probably yes	91	45.27%			
	Probably not	31	15.42%			
	Definitely not	0	0.00%			
Speech and language therapists	Definitely yes	64	39.51%	84.57% (137)	92.59% (150)	77.16% (125)
	Probably yes	73	45.06%			
	Probably not	25	15.43%			
	Definitely not	0	0.00%			
	
Clinical psychologists	Definitely yes	11	50.00%	86.36% (19)	95.45% (21)	54.55% (12)
	Probably yes	8	36.36%			
	Probably not	3	13.64%			
	Definitely not	0	0.00%			
Educational psychologists	Definitely yes	3	30.00%	80.00% (8)	90.00% (9)	70.00% (7)
	Probably yes	5	50.00%			
	Probably not	2	20.00%			
	Definitely not	0	0.00%			
Other	Definitely yes	1	14.29%	85.71% (6)	100.00% (7)	100.00% (7)
	Probably yes	5	71.43%			
	Probably not	1	14.29%			
	Definitely not	0	0.00%			

**TABLE 3:** Therapist‐reported selective mutism training need.

Area of SM training	Percentage of whole sample (*n*)	Percentage of Speech and language therapists (*n*)	Percentage of clinical psychologists (*n*)	Percentage of educational psychologists (*n*)	Percentage of ‘other’ professionals (*n*)
SM awareness	39.80% (80)	37.65% (61)	54.55% (12)	50.00% (5)	28.57% (2)
SM diagnosis	37.31% (75)	37.65% (61)	40.91% (9)	20.00% (2)	42.86% (3)
SM intervention	74.63% (150)	73.46% (119)	81.82% (18)	80.00% (8)	71.43% (5)
Intervention for SM and co‐morbid conditions	62.69% (126)	64.20% (104)	63.64% (14)	40.00% (4)	57.14% (4)
Care‐pathways	38.81% (78)	40.12% (65)	31.82% (7)	40.00% (4)	28.57% (2)
Multi‐disciplinary teams	55.72% (112)	59.26% (96)	50.00% (11)	30.00% (3)	28.57% (2)
Other need	4.98% (10)	4.94% (8)	0.00% (0)	10.00% (1)	14.29% (1)

### 3.4 Research Question 3: Who Are the Therapists Providing the Intervention?

Three quarters of the sample (75.12%, *n* = 151) had provided intervention for children with SM (see Table 2). This group consisted of mostly speech and language therapists (82.78%, *n* = 125) followed by clinical psychologists (7.95%, *n* = 12) and educational psychologists (4.64%, *n* = 7). All of those in the ‘other group’ (4.64%, *n* = 7 play therapists, music therapists, counselling psychologists, and a community mental health nurses) provided intervention.

Just under 7% (*n* = 14) of respondents reported that they had not seen a child with SM for an assessment or intervention in the last five years. Almost a fifth of the respondents (19.25%, *n* = 36) who had seen a child with SM did not provide an intervention because SM children are not part of their caseload (*n* = 22) or because these children were seen by another profession (*n* = 14). From the survey responses to open‐ended questions, participants described situations where there was no provision or only partial provision in their locality. One respondent shared the situation in their locality where many professionals may be involved but worked in indirect ways:

*For children and young people with SM known to CAMHS, CAMHS practitioners from various backgrounds can be involved (e.g. mental health nurses, OT, CAMHS practitioners). However, CAMHS are working on other mental health needs, not directly with the SM (usually). Children and young people without other mental health needs/not know to CAMHS currently don't receive any direct service for SM (signposting and training to education is available)* (Respondent 108).

The survey respondents commented on various barriers to provision such as:

- The limited funds and resources

*We have care pathways but given the ongoing massive cuts these aren't always realistic and I often feel that a psychologist would be better placed to support rather than an SLT* (Respondent 154).

- Difficulties with identification and diagnosis

*There is no way [sic.] robust model for children to even access a formal diagnosis* (Respondent 114).

- No care pathway for SM

*Very inconsistent‐ usually parents and teachers [provide the support for children with SM]. Sometimes speech and language therapy, sometimes educational psychology. School tend to approach one service rather than looking for multiple professionals. No set protocol in our LA [Local Authority]* (Respondent 93).

- No training in SM

*No formal pathway or training makes it very difficult to provide effective intervention and therefore the bulk of my intervention would be advice based to parents and teachers* (Respondent 114).

- Inconsistent service provision across the UK

*No profession currently takes responsibility for these children and young people. Massive postcode lottery. Huge issue* (Respondent 180).

Thirty‐two practitioners reported working in a multi‐disciplinary way with the core professionals consisting of specialists in communication, psychology and education (e.g. speech and language therapists, teachers/education staff, educational psychologists, clinical psychologists/CAMHS). The most frequently reported multi‐disciplinary teams were:

- speech and language therapist, educational psychologist and teacher/education staff (*n* = 9)
- speech and language therapist, CAMHS and teacher/education staff (*n* = 7)
- speech and language therapist, clinical psychologist and teacher/education staff (*n* = 7)

Other professions reported to be involved in the child's intervention included medical professionals (e.g. paediatrician, psychiatrist, mental health nurse), allied health professionals (e.g. occupational therapist, physiotherapist, music therapist), or other mental health professionals (e.g. play therapist, counsellor). One speech and language therapist shared their desire for more collaborative working with this clinical population:

*It would be helpful to work more jointly with psychological services due to the anxiety factor of SM* (Respondent 138).

### 3.5 Research Question 4: What Interventions Are the Therapists Providing?

The respondents providing interventions for children with SM (*n* = 151) most often worked with parents/carers (95%, *n* = 144) and teaching staff (96%, *n* = 145) with the interventions carried out mostly within schools (89%, *n* = 130) (see Table 4).

**TABLE 4:** Preschool and primary school age selective mutism intervention.

Intervention category	Percentage (*n*)
**Who is involved**	
Parent/carer	95% (144)
Teaching staff	96% (145)
Peers	37% (56)
Siblings	25% (38)
**Where is it carried out**	
School	89% (130)
Home	31% (45)
Clinic	33% (48)
Wider community	9% (14)
**Mode of intervention**	
In person only	53% (80)
Online only	7% (11)
Telephone only	<1% (1)
Online and telephone	6% (9)
Combination (in person, online and/or telephone)	33% (50)
**Format of intervention sessions** ^a^	
Training only	1% (2)
Training and indirect	32% (48)
Training and direct	2% (3)
Indirect only	22% (33)
Indirect and direct	10% (15)
Direct only	1% (2)
Combination (training, indirect & direct)	31% (47)

Dosage of intervention	Median	1st Quartile / 3rd Quartile	Interquartile range	Range
Length of sessions (mins)	45	30/55	25	15–180
Number of sessions	6	4/10	6	1–20
Number of weeks	12	10.5/26	15.5	1–200

^a^ **Training** refers to delivering taught sessions on selective mutism to parents/carers and/or education staff;

**Direct intervention sessions** refer to working directly with the child with selective mutism;

**Indirect intervention sessions** refer to working through other people (e.g. parents/carers, education staff) to help the child with selective mutism.

Half of the interventions were carried out in person only (53%, *n* = 80) and a third used a combination of in person, online and telephone support (33%, *n* = 50). Most interventions involved training parents and/or education staff and working indirectly with the child, with just under a third providing training and indirect work (32%, *n* = 48). A similar proportion of the sample provided a combination of training, indirect and direct intervention (31%, *n* = 47) while a fifth provided indirect intervention only (22%, *n* = 33) (see Table 4).

There was extensive variability in the dosage of the interventions provided. We therefore used the median to describe the average length of the session, the number of sessions, and duration of the intervention in weeks for the sample. The medians for each of these metrics were 45 min, six sessions, across twelve weeks respectively (see Table 4).

**FIGURE 2:**
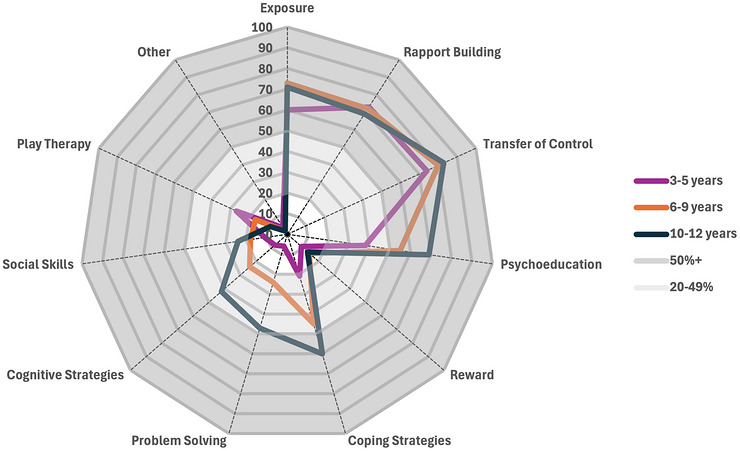
The intervention components used for children 3–5, 6–9, and 10–12 years with selective mutism. *Note*: This figure demonstrates the percentage of therapists who use a particular component when working with children with selective mutism according to age group. Components in the dark grey range of the figure depict the more common components used by therapists (more than 50%). More components are used as age increases. For specific percentages see Table S1.

The intervention components used by the practitioners varied according to child age, becoming more complex by incorporating more components with age (see Figure 2 and see Table S1.). The most common intervention components used across the three age bands (3–5, 6–9, 10–12 years) were exposure (59.83%, 73.23%, 71.43%), rapport building (72.65%, 71.65%, 69.39%), transfer of control (73.50%, 80.31%, 82.65%) and psychoeducation (37.61%, 55.12%, 69.39%). Few therapists indicated using ‘Other’ components outside of the list (4.27%, 2.36%, 2.04%). The ‘Other’ components included parent–child relationship, music therapy, augmentative and alternative communication and interventions dealing with co‐morbidity (e.g. communication difficulties in speech or language).

## 4 Discussion

This is the first UK study to investigate the training, and experiences of professionals in SM, and the services available for children with SM in the context of relevant NHS, Local Authority and HSC settings.

Participants volunteered to take part in the survey and were recruited (in part) via specialist networks. This meant a high likelihood of bias for the participants to have a particular interest in SM. Despite this, only 59% had received some form of SM training. Among the participating practitioners, very few received any form of SM training during their professional qualification course. Instead, when training had been received, it was mostly accessed post‐qualification. There is a need for further investigation into the reasons for the lack of training in professional qualification courses and the effect that this may have on service provision for children with SM; indeed, one respondent reported that without a care‐pathway or training it was ‘very difficult to provide effective interventions’. Alternatively, researchers could explore the extent to which professionals would benefit from having this training during their professional qualification course. As described in open text comments, some respondents had to engage in self‐directed learning. Most of the sample indicated that they needed extra training in SM to do their jobs and deliver SM intervention, including in the context of co‐occurring conditions and working in multi‐disciplinary teams. It appears that little has changed in the last 18 years since Keen et al. (2008) reported the inadequacy of SM training in key professional groups.

SM is an anxiety disorder that can impact educational achievement and social communication which brings a potential role for psychological, educational, and communication specialists. Notably, the three most common professions in the survey sample were speech and language therapists (81%), clinical psychologists (11%) and educational psychologists (5%). It also revealed that a diverse workforce provides intervention to children with SM, including music therapists, play therapists, counselling psychologists, and community mental health nurses. There are likely to be more professions not captured in the survey. This diverse range of professions working with SM poses a challenge to ensure consistent training so that evidence‐based interventions are applied, and that equitable services are provided across the UK. Keen et al. (2008) international consensus group agreed that each local service should identify which professionals take the lead in providing an initial intervention, followed by support from a specialist multidisciplinary team if required. However, if the local services do not identify a profession to take the lead or even have a care pathway for children with SM, as described by several respondents in the survey, then service provision gaps may form with no clear accountability and a risk of inconsistent care. For the children with SM with more complex needs or who were making limited progress, Keen et al.’s consensus group recommended escalating to a specialist multidisciplinary team. In the survey, relatively few participants worked in multidisciplinary teams, but those that did most commonly involved psychological, educational and communication specialists in supporting children with SM, with some wider input from other medical, allied health and mental health professionals.

The interventions used by survey respondents varied according to the age of the child with SM, but they generally became more complex by incorporating more components as children got older. The main components used with 3–5 year‐olds were rapport building, transfer of control, and exposure and to a lesser extent psychoeducation, play therapy, and coping strategies. For children 6–9 years of age, the main components were similar but with higher frequency, and the introduction of cognitive strategies (e.g. cognitive restructuring, testing predictions) and problem solving. For children 10–12 years of age, practitioners reported an increasing frequency of coping strategies, problem solving, cognitive strategies as well as social skills training. While Hipolito et al. (2023) systematic review identified the most frequently used components in nonpharmacological childhood SM intervention studies, it did not differentiate which components were used according to the age of the child. Usage as reported in the survey, however, is broadly consistent with the frequently used components revealed in the systematic review. Notably, the use of rewards in clinical practice (survey results) was much lower than in research practice (Hipolito et al. 2023). In contrast, transfer of control (the gradual transfer of knowledge, skills, and methods from the clinician to parent, teacher, or child (Ginsburg et al. 1995, Hipolito et al. 2023) was reported to be consistently used by survey respondents, yet less frequently reported within research study papers (Hipolito et al. 2023). Whether that reflects limitations in study reporting or a genuine difference between research and clinical practice is unclear. However, it is possible that it reflects the fact that survey respondents worked indirectly with the child (or used a combination of direct and indirect work), predominantly delivering SM interventions with parents/carers and school staff. While this common intervention delivery within school settings aligns with expert recommendations (Keen et al. 2008) and the recent evidence base (Hipolito et al. 2023), the infrequent direct work with children differs from the interventions reported in the systematic review. Some may wonder if this difference in indirect work was precipitated by the COVID‐19 pandemic. However, indirect work (i.e. working with parents/carers and education staff who work directly with the child) has been common clinical practice in SM prior to the pandemic (Keen et al. 2008, Johnson and Wintgens 2016, Johnson et al. 2014). In the review, therapists were found to be working directly with the child in twenty of the twenty‐five studies (Hipolito et al. 2023). This discrepancy highlights the need for future research to establish the effectiveness of working indirectly via parents and teachers.

### 4.1 Limitations

There are several limitations inherent to the study's methodology including the risk of sampling bias, recall bias, and response bias. The survey sample is unlikely to be representative of the paediatric therapy population that provides psychological support or speech and language therapy due to the nonprobability snowball sampling method. The high percentage of speech and language therapists in the sample may be an artefact of this methodology. Therefore, caution is needed when interpreting the data. Although the method potentially skews the data, it was chosen to reach the small and potentially hidden population of therapists working with SM. While there is potential for participants to inaccurately remember details about their SM caseload from the last 5 years, the typically infrequent contact with children with SM (median 3–6 in the last 5 years) might mean that they were likely to be memorable. To minimise recall bias and promote consistency in the use of terminology, participants were provided a list of intervention components with definitions. However we note that, while trying to minimise one bias, providing the definitions based on the recent systematic review (Hipolito et al. 2023) may have influenced participants to respond according to what they perceive is best practice based on the existing evidence base rather than what actually happened.

With the results showing that education staff are often involved in multi‐disciplinary support for children with SM, another limitation of the study is that they were not included in the purposive sample. Although educational psychologists were included and the recruitment flyer was shared with Local Authority professionals, other education staff (e.g. specialist teachers) would not have completed the survey due to the emphasis on ‘therapists’, ‘psychological therapy’ and ‘speech and language therapy’. Consequently, we are missing the valuable views and experiences of other education staff. However, research has started to explore the roles of teachers and their views and experiences in supporting children with SM (White and Bond 2022, Williams et al. 2021).

## 5 Conclusion and Implications

Survey participants indicated that SM training in professional registration courses is scarce. The majority of respondents reported the need for further training in SM to be able to do their jobs, particularly in the area of intervention, working with co‐existing conditions, and working in multi‐disciplinary teams. Those who provided interventions showed great variation in dosage. The use of intervention components, who was involved, and where the interventions occurred generally reflected the current evidence base. However, given that many survey respondents had sought extra training in SM, the broader paediatric therapy population may well be less informed about SM interventions due to the general lack of training.

There is a clear need for more SM training in professional qualification courses and accessible cross‐professional specialist SM training for therapists post‐qualification. The development of cross‐profession guidelines and quality standards for professional training and clinical management of children with SM will help promote consistency (and accountability) across services and a more equitable delivery of evidence‐based interventions for children with SM.

## Funding

GH is funded by an NIHR Clinical Doctoral Research Fellowship (NIHR302167). CC receives funds from the NIHR Applied Research Collaboration Oxford and Thames Valley at Oxford Health NHS Foundation Trust and the Oxford Health Biomedical Research Centre. The views expressed are those of the authors and not necessarily those of the NHS, the NIHR, and the Department of Health and Social Care.

## Ethics Statement

Although ethical approval was sought, the Joint Research Office at the University of Oxford classified the study as a survey that did not require a research ethics review. Nevertheless, research integrity practices were applied including informed consent before survey participation.

## Consent

Participant consent was required to participate in this research.

## Conflicts of Interest

The authors declare no conflicts of interest.

## Permission to reproduce material from other sources

Not presenting material from other sources apart from the authors own ‘Selective mutism intervention components definitions’ as published in their systematic review.

## Supporting information




**Supporting Information**: jlcd70297‐supp‐0001‐SuppMat.docx

**Supporting Information**: jlcd70297‐supp‐0002‐SuppMat.docx

**Supporting Information**: jlcd70297‐supp‐0003‐SuppMat.docx

**Supporting Information**: jlcd70297‐supp‐0004‐SuppMat.docx

## Data Availability

The data that support the findings of this study are available on request from the corresponding author.
