# Supplementary material for: Training, Practice and Service Provision for Selective Mutism: Findings From a UK‐Wide Survey of Therapists
Source: Int J Lang Commun Disord. 2026 Aug 10;61(5):e70297. doi: 10.1111/1460-6984.70297 (PMC13456093; doi:10.1111/1460-6984.70297)
Supplement: Supplementary file 1 [file JLCD-61-0-s001.docx]

**Supplementary Materials 1 Thirty-two item questionnaire used in the study**

The 32 items consist of questions one to 23 including sub-questions (a-c and i-vi for certain questions).

**Part 1: Understanding paediatric speech and language therapists’ and psychological therapists’ (e.g. CAMHS workers, clinical psychologists, educational psychologists, play therapists, music therapists, Psychological and well-being practitioners) awareness, training, and experience of SM in the UK. (16 questions timing - 4 minutes)**

1) What is your main profession when working with children 3 to 12 years of age?

❑Art therapist

❑Children’s well-being practitioner

❑Community mental health nurse

❑Clinical psychologist

❑Educational mental health practitioner

❑Educational psychologist

❑Music therapist

❑Play therapist

❑Psychotherapist

❑Social worker

❑Speech and language therapist

❑Other: (open text)_________

2) How long have you worked in this profession? (to the nearest whole year)
3) Which UK country do you work in?

❑ England

❑ Northern Ireland

❑ Scotland

❑ Wales

4) What is your age? (to the nearest whole year)
5) What is your highest level of education?

❑College (e.g. A level/ Diploma)

❑Undergraduate (e.g. BA, BSc)

❑Pre-registration (professional qualification) postgraduate diploma/masters

❑Post-registration postgraduate certificate/diploma

❑Post-registration masters

❑Doctorate (including clinical doctorate, PhD, professional doctorate)

6) How would you rate your knowledge and understanding of selective mutism? (where 1 is no knowledge or understanding of SM and 7 is expert knowledge and understanding of SM)

❑ 1

❑ 2

❑ 3

❑ 4

❑ 5

❑ 6

❑ 7

7) Please answer the next 6 questions either True, Don’t know or False?

7a) A child with selective mutism chooses not to speak.

❑True ❑ Don’t know ❑False

7b) Most cases of selective mutism are caused by trauma.

❑True ❑ Don’t know ❑False

7c) A child with selective mutism is unable to speak in specific social situations where talking is expected while being able to speak freely in other situations.

❑True ❑ Don’t know ❑False

7d) Selective mutism is considered an anxiety disorder.

❑True ❑ Don’t know ❑False

7e) A child with selective mutism could also have a diagnosis of autism.

❑True ❑ Don’t know ❑False

7f) A bilingual or multilingual child is more likely to develop selective mutism than a monolingual child.

❑True ❑ Don’t know ❑False

8) Have you received any training on selective mutism? ❑Yes ❑No
9) If yes, When did you receive this training (multiple answers allowed)

❑College qualification (e.g. diploma)

❑Undergraduate Degree (e.g. BA, BSc)

❑Pre-registration (professional qualification) postgraduate diploma/masters

❑Post-registration postgraduate certificate/diploma

❑Post-registration masters

❑Doctorate (including clinical doctorate, PhD, professional doctorate)

❑Additional specialist training post qualification training

❑Inset/In-house training on SM

9b) What was the name of the specific module, lecture or course during your training?

9c) How many hours of selective mutism training did you receive during your training? (to the nearest whole hour)

If no to Q8, proceed to Q10.

10) Do you think you need any extra training in selective mutism to do your job?

❑Definitely not

❑Probably not

❑Probably yes

❑Definitely yes

[If Definitely not or Probably not, proceed to Q12]

11) In what area(s) would you find training in selective mutism helpful? (multiple answers allowed)

❑SM awareness (prevalence, risk factors, causes, general research)

❑Diagnosis

❑Intervention approaches

❑Treating SM with comorbidity. Which conditions? (open text) _______

❑Care pathways

❑Multi-disciplinary teams working with SM

❑Other: (open text) ______

**Answering this question is required to move on.**

12) Approximately how many children with SM have you seen in the last 5 years (either for **assessment** or **intervention**)?

❑None

❑1-2

❑3-6

❑7-12

❑13-20

❑21+

[if none, survey ends, if 1 or more, continue]

**Answering this question is required to move on.**

13) Approximately, how many children with selective mutism have you provided **intervention** for in the last 5 years?

❑None

❑1-2

❑3-6

❑7-12

❑13-20

❑21+

[If none,]

13b) Is this because selective mutism (SM) is not part of your caseload or are children with SM seen by team members from another professional background? (then close survey)

❑Not part of caseload

❑Selective mutism seen by team members from another professional background. Which profession(s): (open text) ______

[If 1 or more, continue to **Part 2**]

**Part 2: Understanding your intervention Understanding the interventions for children with selective mutism (3-12 years) in the UK provided by therapists in NHS and Local Authority services. (11 questions timing - 8 minutes)**

14) What is the typical format of the intervention you offer: (multiple answers allowed)

❑Delivering training on SM to parents/carers

❑Delivering training on SM to teachers/education staff

❑Indirect therapy/consultation with parents/carers

❑Indirect therapy/consultation with teachers/education staff

❑Direct 1:1

❑Direct group work

❑Other: [open text] _______

15) What is the typical mode of the intervention? (multiple answers allowed)

❑Face to face

❑Online

❑Telephone

16) Which intervention components do you typically use with children with SM ages 3-5 years? (multiple answers allowed)

Click on link for definitions: SM component intervention definitions

❑Don’t see this age group

❑Cognitive strategies (i.e. change in thinking of anxious thoughts)

❑Coping strategies (i.e. equipping child to cope with their anxiety)

❑Exposure (i.e. small steps exposure to situations moving towards speech)

❑Play therapy (i.e. helping child communicate and process emotions and thoughts through play)

❑Problem solving (i.e. equipping child to solve problems linked to SM)

❑Psychoeducation (i.e. learning about SM, what does and doesn’t help)

❑Rapport building (i.e. adapting interaction style to build rapport and facilitate communication with child)

❑Reward/Contingency management (i.e. positive reinforcement for appropriate communication behaviours)

❑Social skills (i.e. equipping child with skills to improve interaction)

❑Transfer of control (i.e. empowering parents, teachers, and/or child to implement intervention outside of sessions)

❑ Other [open text] _____

17) Which intervention components do you typically use with children with SM ages 6-9 years? (multiple answers allowed)

Click on link for definitions: SM component intervention definitions

❑Don’t see this age group

❑Cognitive strategies (i.e. change in thinking of anxious thoughts)

❑Coping strategies (i.e. equipping child to cope with their anxiety)

❑Exposure (i.e. small steps exposure to situations moving towards speech)

❑Play therapy (i.e. helping child communicate and process emotions and thoughts through play)

❑Problem solving (i.e. equipping child to solve problems linked to SM)

❑Psychoeducation (i.e. learning about SM, what does and doesn’t help)

❑Rapport building (i.e. adapting interaction style to build rapport and facilitate communication with child)

❑Reward/Contingency management (i.e. positive reinforcement for appropriate communication behaviours)

❑Social skills (i.e. equipping child with skills to improve interaction)

❑Transfer of control (i.e. empowering parents, teachers, and/or child to implement intervention outside of sessions)

❑ Other [open text] _____

18) Which intervention components do you typically use with children with SM ages 10-12 years? (multiple answers allowed)

Click on link for definitions: SM component intervention definitions

❑Don’t see this age group

❑Cognitive strategies (i.e. change in thinking of anxious thoughts)

❑Coping strategies (i.e. equipping child to cope with their anxiety)

❑Exposure (i.e. small steps exposure to situations moving towards speech)

❑Play therapy (i.e. helping child communicate and process emotions and thoughts through play)

❑Problem solving (i.e. equipping child to solve problems linked to SM)

❑Psychoeducation (i.e. learning about SM, what does and doesn’t help)

❑Rapport building (i.e. adapting interaction style to build rapport and facilitate communication with child)

❑Reward/Contingency management (i.e. positive reinforcement for appropriate communication behaviours)

❑Social skills (i.e. equipping child with skills to improve interaction)

❑Transfer of control (i.e. empowering parents, teachers, and/or child to implement intervention outside of sessions)

❑ Other [open text] _____

19) Do children with selective mutism typically receive a multi-disciplinary approach within your service? ❑Yes ❑No

[If yes, answer Q20. If no, proceed to Q21]

20) Which other professions are involved in the multi-disciplinary service? [open text] ______
21) Who else is involved in the intervention? (multiple answers allowed)

❑Parents/Carers

❑Teachers/Education staff

❑Siblings

❑Peers

❑Other: [open text] ________

22) Where does the intervention typically take place? (multiple answers allowed)

❑Clinic/Office

❑Home

❑School

❑Community (e.g. shops, street, restaurant)

❑Other: [open text] _______

23a) On average, taking into account the last 3 children with SM you saw, ***what is the length of a session*** that you deliver to children with selective mutism? (average length of time in minutes)

23b) On average, taking into account the last 3 children with SM you saw, ***how many sessions*** are included in a package of care or treatment plan for a child with selective mutism? (average number of sessions in whole numbers)

23c) On average, taking into account the last 3 children with SM you saw, what is the typical ***length of a package of care or treatment plan*** for a child with selective mutism? (average length of time in weeks)

**Thank you for completing the survey.**
