# Supplementary material for: Training, Practice and Service Provision for Selective Mutism: Findings From a UK‐Wide Survey of Therapists
Source: Int J Lang Commun Disord. 2026 Aug 10;61(5):e70297. doi: 10.1111/1460-6984.70297 (PMC13456093; doi:10.1111/1460-6984.70297)
Supplement: Supplementary file 3 [file JLCD-61-0-s003.docx]

**Supplementary Materials 3 Data Integrity Protocol v4Feb24**

This Data Integrity Protocol is the plan for preventing, identifying and removing fraudulent survey responses before the study analysis. The protocol also provides guidelines to identify responses that are duplicates or deemed incomplete. The protocol involves a two stepped approach to maintain sample validity and data integrity:

- Implementing preventative measures in the study design
- Reviewing the final survey responses with two coders to remove incomplete surveys, duplicates and responses that are “high risk” of fraud.

**Preventative measures in the study design**

The following preventative measures were implemented in the study design

- Remove terms “survey” or “study” from the text of the survey weblink (therefore, weblink is bit.ly/PRISM_therapistUK) (Wang et al., 2023).
- Keep default IP logging and location data features in Qualtrics to indicate possible fraudulent responses (Griffin et al., 2022; Pozzar et al., 2020; Wang et al., 2023).
- Use Qualtrics Prevent Multiple Submissions by enabling ‘flag responses’. These flagged responses activate a Q_BallotBoxStuffing score (Qualtrics, 2023). Some participants may make a mistake in their submitted survey and therefore repeat the survey. However, repeated survey submissions may also be an indicator of fraudulent responses.
- Use Qualtrics Bot Detection which activates a Q_RecaptchaScore (Qualtrics, 2023).
- Enabling Security Scan Monitor in Qualtrics to prevent email scanning software from starting a survey session (Qualtrics, 2023).
- Enabling RelevantID in Qualtrics which creates four different scores: RelevantIDDuplicate; RelevantIDDuplicateScore; Relevant IDFraudScore; and RelevantIDLastStartDate (Qualtrics, 2023).
- No financial incentives for survey completion (Pozzar et al., 2020).

**Reviewing final survey responses**

The reviewing process involves two coders of the research team (GH and RB) to assess the data integrity of the survey responses before analysis. If the two coders are not in agreement, a third member of the research team (CC) will be consulted to make the final decision. The steps for reviewing the data are:

- Check Inclusion/Exclusion criteria
- Check Finished status (less than 60% completion)
- Check Completion time
- Check Duplicate IP address (go into column- choose sort ascending or descending order- click undo afterwards or data will be compromised)
- Check Q_BallotBoxStuffing
- Check Q_RelevantIDDuplicateScore
- Check Q_RelevantIDFraudScore
- Check Q_RecaptchaScore
- Check inconsistency or irrelevant content (age/length time in profession)(Q13 number exceeding Q12)
- Check exact response in open ended questions (**see Open ended questions list**)
- Check incoherent text or unrelated answer in open-ended question (**see Open ended questions list**)

Respondents who did not fulfil inclusion criteria and responses with less than 60% completion **(see Algorithm for completion rate below)** will score 2pts and therefore be removed before the final data analysis (Griffin et al., 2022). The survey responses will be judged on the following criteria of **Table 1**. derived from other studies using data integrity protocols for online surveys (Griffin et al., 2022; Pozzar et al., 2020; Wang et al., 2023).

**Table 1. Indicators of duplication and possible fraud for the PRISM therapist survey.**

| **Point value** | **Indicators of possible fraud** |
| --- | --- |
| 1 pt | Completion time infeasibility (< 4 minutes up to Q22; < 1 minute 50 seconds up to Q12/Q13) based on study pilot times. (Griffin et al., 2022; Wang et al., 2023). |
| 1 pt | Duplicate IP Addresses  Scoring True (1) in Q_BallotBoxStuffing or Q_RelevantIDDuplicate score and/or scoring 75 or more in the Q_RelevantIDDuplicateScore (Qualtrics, 2023). These scores would indicate duplicate IP addresses which are at risk of being fraudulent responses (Griffin et al., 2022; Pozzar et al., 2020 ; Wang et al., 2023). Then check Q_RelevantIDLastStart to find matching IP Addresses. Since therapists working in the NHS, Local Authority or Health and Social Care settings may use the same computer in the office, it may trigger the at-risk scores. Therefore, further review of the responses is needed. **Duplicate Rule:** If respondents have the same IP address and the same demographic information, keep the last appropriate response (i.e. not scoring anymore points) and delete the other copies. This is to accommodate a participant who tries to complete the survey again after reflecting on their previous responses. |
| 1 pt | Q_RelevantIDFraudScore is equal to or greater than 30 means the response is at risk of being fraudulent and a bot (Qualtrics, 2023). |
| 2 pt | Q_RelevantIDFraudScore is 130 (maximum value – highly likely to be fraudulent) |
| 1 pt | Q_RecaptchaScore is less than 0.5 means the response is at risk of being a bot (Qualtrics, 2023). |
| 2 pts | Q_RecaptchaScore is 0 (minimum value – highly likely to be a bot) |
| 1 pt | Inconsistency or irrelevant content in data reported within the survey (e.g. length of time in the profession and age, number of cases seen for intervention Q13 exceeds number of total selective mutism cases seen Q12) (Griffin et al., 2022; Pozzar et al., 2020; Wang et al., 2023). |
| 1 pt | Exact response (consisting of more than 3 words) provided by more than one respondent in open ended items (Pozzar et al., 2020) |
| 1 pt | Incoherent text or unrelated answer in open-ended question (Pozzar et al., 2020; Wang et al., 2023). |
| **Any survey responses that score on any of the above, their survey location will be checked:** | |
| 1 pt | For these surveys completed outside of the UK using Qualtrics location data. |

Any survey response that scores 2 or more is considered to be ‘high risk of fraud’ and therefore will be removed before the final study analysis unless following the Duplicate rule (see Table 1.).

***Open ended questions list***

1_Q9b. What was the name of the specific module, lecture, or course during your training?

2_Q9b. What was the name of the specific module, lecture, or course during your training?

3_Q9b. What was the name of the specific module, lecture, or course during your training?

4_Q9b. What was the name of the specific module, lecture, or course during your training?

5_Q9b. What was the name of the specific module, lecture, or course during your training?

6_Q9b. What was the name of the specific module, lecture, or course during your training?

7_Q9b. What was the name of the specific module, lecture, or course during your training?

15_Q9b. What was the name of the specific module, lecture, or course during your training?

Q11_4_Text. In what area(s) would you find training in selective mutism helpful? Treating SM with comorbidity. Which conditions?

Q11_7_Text. In what area(s) would you find training in selective mutism helpful? Other: Text

Q13b_2_Text. Is this because SM is not part of your caseload or are children with SM seen by team members from another professional background?

Q14_5_Text. What is the typical format of the intervention you offer? Other: Text

Q16_12_Text. Which intervention components do you typically use with children with SM aged 3-5 years?

Q17_12_Text. Which intervention components do you typically use with children with SM aged 6-9 years?

Q18_12_Text. Which intervention components do you typically use with children with SM aged 10-12 years?

Q20. Which professions are involved in the multi-disciplinary service?

Q21_5_Text. Who else is involved in the interventions?

Q22_5_Text. Where does the intervention typically take place? Other: Text

Q23a. … what is the length of a session that you deliver to children with selective mutism?

Q23b. … how many sessions are included in a package of care or treatment plan for a child with selective mutism?

Q23c … what is the typical length of a package of care or treatment plan for a child with selective mutism?

***Algorithm for completion rate***

The 60% completion rate will be determined by the Qualtrics Progress field or the following manual algorithm:

Questions included in the percentage calculated as completed:

1, 2, 3, 4, 5, 6, 7a, 7b, 7c, 7d, 7e, 7f, 8, 9, (9b), (9c), 10, 11, 12, 13a, (13b), 14, 15, 16, 17, 18, 19, 20, 21, 22, 23a, 23b, 23c.

60% based on 17-32 possible questions asked in the survey:

16 questions if person answers Q8 as “no” and Q12 as “none”. Therefore, 60% is 10 questions.

18 questions if person answers Q8 as “no” and Q13a as “none”. Therefore, 60% is 11 questions.

19 questions if person answers Q8 as “yes” and Q12 as “none”. Therefore, 60% is 12 questions.

21 questions if person answers Q8 as “yes” and Q13a as “none”. Therefore, 60% is 13 questions.

29 questions if person answers Q8 as “no” and Q13a as 1 or more. Therefore, 60% is 18 questions.

32 questions if person answers Q8 as “yes” and Q13a as 1 or more. Therefore, 60% is 20 questions.

**References**

Griffin, M., Martino, R. J., LoSchiavo, C., Comer-Carruthers, C., Krause, K. D., Stults, C. B., & Halkitis, P. N. (2022). Ensuring survey research data integrity in the era of internet bots. *Qual Quant*, *56*(4), 2841-2852. <https://doi.org/10.1007/s11135-021-01252-1>

Pozzar, R., Hammer, M. J., Underhill-Blazey, M., Wright, A. A., Tulsky, J. A., Hong, F., Gundersen, D. A., & Berry, D. L. (2020). Threats of Bots and Other Bad Actors to Data Quality Following Research Participant Recruitment Through Social Media: Cross-Sectional Questionnaire. *J Med Internet Res*, *22*(10), e23021. <https://doi.org/10.2196/23021>

Qualtrics. (2023). *Fraud Detection*. Retrieved 02 October from <https://www.qualtrics.com/support/survey-platform/survey-module/survey-checker/fraud-detection/?utm_medium=product&utm_source=survey-builder#BotDetection>

Wang, J., Calderon, G., Hager, E. R., Edwards, L. V., Berry, A. A., Liu, Y., Dinh, J., Summers, A. C., Connor, K. A., Collins, M. E., Prichett, L., Marshall, B. R., & Johnson, S. B. (2023). Identifying and preventing fraudulent responses in online public health surveys: Lessons learned during the COVID-19 pandemic. *PLOS Glob Public Health*, *3*(8), e0001452. <https://doi.org/10.1371/journal.pgph.0001452>
