# Supplementary material for: Training, Practice and Service Provision for Selective Mutism: Findings From a UK‐Wide Survey of Therapists
Source: Int J Lang Commun Disord. 2026 Aug 10;61(5):e70297. doi: 10.1111/1460-6984.70297 (PMC13456093; doi:10.1111/1460-6984.70297)
Supplement: Supplementary file 4 [file JLCD-61-0-s004.docx]

**Table S1 Intervention components used by practitioners according to child age**

| **Intervention Components** | **3-5 years**  **Percentage (n=117)** | **6-9 years**  **Percentage (n=127)** | **10-12 years**  **Percentage (n=98)** |
| --- | --- | --- | --- |
| Exposure | 59.83% (70) | 73.23% (93) | 71.43% (70) |
| Reward/Contingency management | 9.40% (11) | 14.17% (18) | 13.27% (13) |
| Psychoeducation | 37.61% (44) | 55.12% (70) | 69.39% (68) |
| Rapport building | 72.65% (85) | 71.65% (91) | 69.39% (68) |
| Transfer of control | 73.50% (86) | 80.31% (102) | 82.65% (81) |
| Coping strategies | 20.51% (24) | 44.88% (57) | 60.20% (59) |
| Social skills | 11.11% (13) | 18.11% (23) | 24.49% (24) |
| Cognitive strategies | 7.68% (9) | 24.41% (31) | 41.84% (41) |
| Problem solving | 5.98% (7) | 24.41% (31) | 46.94% (46) |
| Play therapy | 27.35% (32) | 16.54% (21) | 9.18% (9) |
| Other | 4.27% (5)^a^ | 2.36% (3)^b^ | 2.04% (2)^c^ |

Shading: 20-49%; 50% or more.

^a^ 3-5 years, Other components included: Parent-Child Relationship; Music Therapy; Augmentative and Alternative Communication; Intervention Dealing with Co-morbidity e.g. communication difficulties.

^b^ 6-9 years, Other components included: Interventions Dealing with Co-morbidity e.g. communication difficulties; Augmentative and Alternative Communication.

^c^ 10-12 years, Other components included: Interventions Dealing with Co-morbidity e.g. communication difficulties; Augmentative and Alternative Communication.
