# Supplementary material for: Training, Practice and Service Provision for Selective Mutism: Findings From a UK‐Wide Survey of Therapists
Source: Int J Lang Commun Disord. 2026 Aug 10;61(5):e70297. doi: 10.1111/1460-6984.70297 (PMC13456093; doi:10.1111/1460-6984.70297)
Supplement: Supplementary file 5 [file JLCD-61-0-s002.docx]

**Supplementary Materials 2 Selective mutism intervention component definitions**
